# Causal associations between prostate diseases, renal diseases, renal function, and erectile dysfunction risk: a 2-sample Mendelian randomization study

**DOI:** 10.1093/sexmed/qfae002

**Published:** 2024-02-10

**Authors:** Diliyaer Dilixiati, Kaisaierjiang Kadier, Jian-De Lu, Shiping Xie, Baihetiya Azhati, Reyihan Xilifu, Mulati Rexiati

**Affiliations:** Department of Urology, First Affiliated Hospital of Xinjiang Medical University, Urumqi 830054, China; Department of Cardiology, First Affiliated Hospital of Xinjiang Medical University, Urumqi 830054, China; Department of General Surgery, Children's Hospital of Xinjiang Uygur Autonomous Region, Urumqi 830010, China; Department of Urology, First Affiliated Hospital of Xinjiang Medical University, Urumqi 830054, China; Department of Urology, First Affiliated Hospital of Xinjiang Medical University, Urumqi 830054, China; Department of Nephrology, Children's Hospital of Xinjiang Uygur Autonomous Region, Urumqi 830054, China; Department of Urology, First Affiliated Hospital of Xinjiang Medical University, Urumqi 830054, China

**Keywords:** prostate diseases, renal diseases, prostate cancer, chronic renal disease, erectile dysfunction, Mendelian randomization

## Abstract

**Background:**

Previous observational studies have found a potential link between prostate disease, particularly prostate cancer (PCa), and kidney disease, specifically chronic renal disease (CKD), in relation to erectile dysfunction (ED), yet the causal relationship between these factors remains uncertain.

**Aim:**

The study sought to explore the potential causal association between prostate diseases, renal diseases, renal function, and risk of ED.

**Methods:**

In this study, 5 analytical approaches were employed to explore the causal relationships between various prostate diseases (PCa and benign prostatic hyperplasia), renal diseases (CKD, immunoglobulin A nephropathy, membranous nephropathy, nephrotic syndrome, and kidney ureter calculi), as well as 8 renal function parameters, with regard to ED. All data pertaining to exposure and outcome factors were acquired from publicly accessible genome-wide association studies. The methods used encompassed inverse variance weighting, MR-Egger, weighted median, simple mode, and weighted mode residual sum and outlier techniques. The MR-Egger intercept test was utilized to assess pleiotropy, while Cochran’s Q statistic was employed to measure heterogeneity.

**Outcomes:**

We employed inverse variance weighting MR as the primary statistical method to assess the causal relationship between exposure factors and ED.

**Results:**

Genetically predicted PCa demonstrated a causal association with an elevated risk of ED (odds ratio, 1.125; 95% confidence interval, 1.066-1.186; *P <* .0001). However, no compelling evidence was found to support associations between genetically determined benign prostatic hyperplasia, CKD, immunoglobulin A nephropathy, membranous nephropathy, nephrotic syndrome, kidney ureter calculi, and the renal function parameters investigated, and the risk of ED.

**Clinical Implications:**

The risk of ED is considerably amplified in patients diagnosed with PCa, thereby highlighting the importance of addressing ED as a significant concern for clinicians treating individuals with PCa.

**Strengths and Limitations:**

This study’s strength lies in validating the PCa-ED association using genetic analysis, while its limitation is the heterogeneity in study results.

**Conclusion:**

The results of this study suggest a potential link between PCa and a higher risk of ED.

## Introduction

Erectile dysfunction (ED) is a condition characterized by the inability to achieve or sustain a satisfactory penile erection during sexual intercourse.^1^ ED has been observed to be more prevalent in middle-aged and elderly populations, with the likelihood of experiencing it increasing as individuals age.^2^ However, there is a growing concern about the rising prevalence of ED among young men, with estimates suggesting that it may affect up to 30% of this population.^3^ The etiology and pathogenesis of ED are highly complex and are generally believed to be associated with unhealthy lifestyles, chronic diseases, and psychological factors.^4^

Among the various men’s health topics on the Internet, prostate disease and ED are among the most popular search topics. This clearly indicates the public’s significant concern regarding these particular conditions.^5^ In the field of urology and andrology medicine, several previous studies have indicated a correlation between prostate disease and ED,^6^^,^^7^ suggesting the presence of shared underlying factors.^8^ In patients with prostate cancer (PCa), the presence of inflammation and increased levels of reactive oxygen species are linked to reduced blood flow necessary for achieving a penile erection.^9^ In addition, various treatment options currently available, such as radical prostatectomy and radiotherapy, carry the risk of causing ED.^10^ This makes it difficult to directly study the relationship between prostate disease and ED. At the same time, it has been discovered that kidney disease, which is also a urinary condition, is linked to ED. Meta-analysis has revealed a high prevalence of ED in individuals with end-stage renal disease,^11^ and it has been found that treatments such as kidney transplantation can significantly improve erectile function.^12^ Due to the potential shared microvascular pathophysiological pathways between renal disease and ED, the interaction between these 2 conditions is complex and bidirectional.^13^ However, it is crucial to acknowledge that a considerable number of these studies were cross-sectional analyses, which possess certain limitations in terms of methodological consistency, heterogeneity, and potential underrepresentation. Additionally, it is important to highlight that no definitive causal association between these diseases has been established.

Mendelian randomization (MR) has emerged as a widely utilized method for causal inference. By leveraging germline genetic variation as an instrumental variable, this approach significantly mitigates the potential for bias stemming from reverse causation and residual confounding factors.^14^ Consequently, MR enables the identification of robust and impactful causal associations. The application of MR tools enables the validation of existing cross-sectional study associations, thereby establishing causal relationships and discerning the directional causality between diseases. Furthermore, our study signifies the pioneering investigation into the potential correlation between partial measures of renal function and ED. Our research has contributed to the ongoing validation of key risk factors for ED, and has aimed to modestly raise awareness among clinicians and preventive medicine practitioners regarding the prevalence of this condition. Through our findings, we hope to modestly support the implementation of targeted etiological prevention measures, potentially contributing to a reduction in the overall risk of developing ED.

Eliminating the influence of confounding factors is a significant challenge for current observational studies, thereby hindering the ability to infer a cause-and-effect relationship between prostate disease, kidney disease, and ED. By using MR analysis, we can overcome this limitation and obtain more reliable results based on available data.^15^ Considering the risk factors of ED, such as advancing age, type 2 diabetes, smoking, and other relevant factors, it becomes evident that these diseases and disease indicators are significantly influenced. Does the potential association between prostate diseases, renal diseases, renal function, and ED remain independent and causal?

## Methods

### Study design

This study employs a 2-sample MR design utilizing summary statistics from genome-wide association studies (GWASs) and extensive biobanks. The utilization of MR analysis facilitates the assessment of causality between exposure and outcome.^16^ Considering the association between PCa and ED serves as a prime illustration. Within cross-sectional studies of PCa patients, the treatment options, such as radical prostatectomy and radiation therapy are frequently administered, potentially leading to the rapid onset of ED. The diverse treatment modalities available, such as radical prostatectomy and radiotherapy,^17^ carry a potential risk of inducing ED themselves. Furthermore, the procedure for confirming the diagnosis, such as prostate puncture, may also contribute to the development of ED. These factors pose challenges to the examination of causal links between PCa and ED within current cross-sectional studies, thereby emphasizing the suitability of MR methods for such explorations.

We obtained data from the Integrative Epidemiology Unit (IEU) OpenGWAS database, which was curated by the Medical Research Council IEU based at the University of Bristol (https://gwas.mrcieu.ac.uk/).^18^ Participants provided written informed consent in previous studies approved by ethical review boards. As the data used were from public databases, no additional ethical approval was required. Details pertaining to study characteristics, participant information, and ethical statements for each dataset were extracted meticulously from the respective original publications or websites. Additionally, this study adhered to the STROBE-MR (Strengthening the Reporting of Observational Studies in Epidemiology using Mendelian Randomization) guideline, which enhances the reporting of MR studies in epidemiology.^19^

**Figure 1:**
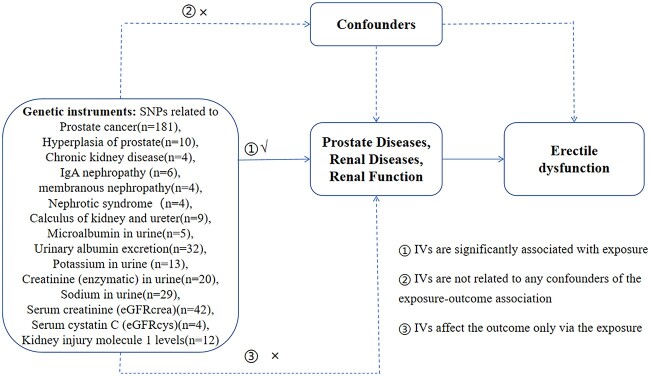
Schematic diagram of the Mendelian randomization design, rationale, and procedures.

The prostate diseases assessed in this study encompassed PCa and benign prostatic hyperplasia (BPH), while the renal diseases encompassed CKD, immunoglobulin A (IgA) nephropathy, membranous nephropathy, nephrotic syndrome, and kidney and ureter calculi. Additionally, the renal function parameters assessed in this study encompassed enzymatic creatinine in urine, urinary albumin excretion, microalbumin in urine, potassium in urine, sodium in urine, serum creatinine (eGFRcrea), serum cystatin C (eGFRcys), and levels of kidney injury molecule 1. Considering the primary focus of our study on prostate disease and kidney disease, we incorporated and analyzed all associated conditions. However, it is important to note that certain diseases such as prostatitis, renal cell carcinoma, congenital kidney malformations, and others experienced a dearth of available single nucleotide polymorphisms (SNPs) in the existing published public data. Consequently, we were unable to include these particular diseases in our study due to insufficient genetic information. Furthermore, all study participants were of European ancestry, ensuring that there was no sample overlap between the exposure and outcome traits. The overview of our MR analyses is shown in Figure 1.

### Data source

The PCa GWAS data utilized in this study were derived from a meta-analysis that integrated findings from 7 preceding PCa GWAS datasets.^20^ In total, the meta-analysis encompassed a sample size of 140 254 individuals, comprising 79 148 cases and 61 106 controls. The pooled GWAS data for BPH were obtained from round 7 of the FinnGen consortium. The study utilized samples from the Finnish Biobank, comprising a total of 13 118 cases and 72 799 controls.^21^

To investigate various renal diseases, such as CKD, IgA nephropathy, membranous nephropathy, nephrotic syndrome, and kidney ureter calculi, we curated European ancestry sample populations sourced from publicly available data and the FinnGen study that have been previously published.^22-24^ The extensive dataset curated by the UK Biobank served as the source for the GWAS data pertaining to microalbumin in urine, enzymatic creatinine in urine, and sodium in urine. This valuable resource comprises an impressive cohort of approximately 500 000 participants from the United Kingdom, which provides a robust foundation for our analyses.^25^ In addition, the GWAS data for urinary albumin excretion, estimated glomerular filtration rate (eGFR), and potassium excretion in urine were acquired from a merged sample dataset derived from the UK Biobank database.^26-28^ Moreover, the GWAS data for serum cystatin C, kidney injury molecule 1 levels, and serum creatinine are encompassed as part of the CKDGen consortium.^29^

The ED GWAS dataset, the largest of its kind for studying ED in individuals of European ancestry, combined 3 cohorts and recruited a total of 223 805 subjects (6175 cases and 217 630 controls).^30^

### Genetic instrument selection

We employed stringent criteria based on the European 1000 Genomes panel to extract SNPs associated with exposure factors. First, we considered SNPs that demonstrated genome-wide significance (*P* < 5 × 10^−8^) and exhibited independence (r^2^ < 0.001) from other SNPs, with a clumping distance of 10 000 kb. Second, as part of the harmonization process, the palindromic SNPs were excluded from the instrumental variables (IVs). Third, to ensure that the IVs exclusively influence the risk of ED through exposure factors, we conducted a comprehensive analysis using the PhenoScanner database (http://www.phenoscanner.medschl.cam.ac.uk/). We carefully examined and removed any potentially confounding factor-related SNPs associated with increasing age, type 2 diabetes, metabolic syndrome, smoking, anxiety, depression, bipolar disorder, sleep disorder, and insomnia. These confounding risk factors were identified based on current guidelines for ED^31^ and by referencing published articles on MR.^32^ Finally, variables with *F* values <10, which serve as indicators of IV intensity, were excluded in order to minimize potential bias resulting from weak instrumental detection.

### Statistical analyses

We conducted 2-sample MR analyses to evaluate the causal associations between prostate disease, renal disease, renal function, and ED. This MR study was undertaken based on 3 fundamental premises: (1) the strong association of genetic variation with exposure; (2) the absence of any association between genetic variation and potential confounders; and (3) genetic variations are independent of outcome, except by means of exposure. We utilized inverse variance–weighted (IVW) MR as the main statistical method, while also employing weighted median MR and mode-based, MR-Egger, and MR-PRESSO analyses to investigate the relationship between exposure factors and ED. Cochran’s Q test assessed SNP heterogeneity, with *P* < .05 indicating high heterogeneity. Additionally, the intercept term within the MR-Egger regression approach was employed as a means to discern directional pleiotropic effects, with a significance level of *P* < .05 being indicative of the presence of pleiotropy. Moreover, a leave-one-out sensitivity analysis was performed to investigate the potential impact of individual SNPs in introducing bias and affecting the overarching causal effect. The analysis was conducted using the TwoSampleMR package (version 0.5.6) within the R statistical computing environment (version 4.2.1; R Foundation for Statistical Computing). We utilized GraphPad Prism (version 9; GraphPad Software) as our preferred software for generating visual representations. A 2-tailed *P* < .05 was considered statistically significant.

**Ethics and Informed Consent**

In our current study, we solely relied on publicly accessible summary data, and the ethical approval as well as consent from participants were obtained through the original GWAS. Each of the studies contributing to the GWAS had obtained informed consent from study participants.

## Results

### Selected genetic instruments (IVs)

Detailed information regarding the 5 methods employed can be found in Supplementary Table 1. Subsequent to the exclusion of LD SNPs (r^2^ > 0.001 within 10 000 kb), palindromic SNPs, duplicated SNPs, and weak-effect SNPs (*P* > 5 × 10^−8^ or *F* < 10), we manually eliminated SNPs associated with potential confounders such as increasing age, type 2 diabetes, metabolic syndrome, smoking, anxiety, depression, bipolar disorder, sleep disorder, and insomnia. Five SNPs used for genetic prediction of PCa, 1 for IgA nephropathy, 4 for urinary albumin excretion, 1 for potassium in urine, 3 for sodium creatine in urine, 2 for serum creatinine (eGFRcrea), and 1 for serum cystatin C (eGFRcys) were excluded due to the presence of confounding factors.(Supplementary Tables 2 and 3).

Table 1 presents the essential characteristics of the dataset utilized in this study. In this set of screening criteria, we reserved 118 index SNPs for the genetic prediction of PCa, 10 for BPH, 6 for IgA nephropathy, 4 for membranous nephropathy, 4 for nephrotic syndrome, 9 for kidney and ureter calculi, 5 for microalbumin in urine, 32 for urinary albumin excretion, 13 for potassium in urine, 20 for creatinine in urine, 29 for sodium in urine, 42 for serum creatinine (eGFRcrea), 4 for nephrotic syndrome, 4 for serum cystatin C (eGFRcys), and 12 for levels of kidney injury molecule 1.

**Table 1:** Details of the GWASs included in the Mendelian randomization.

**Trait**	**Consortium**	**Sample size**	**Population**	**GWAS ID**	**PMID**	**nSNP**
Prostate cancer	PRACTICAL	140 254	European	ebi-a-GCST006085	29 892 016	118
Hyperplasia of prostate	FinnGen	85 917	European	finn-b-N14_PROSTHYPERPLA	NA	10
Chronic kidney disease	NIH	117 165	European	ebi-a-GCST003374	26 831 199	4
IgA nephropathy	NIH	477 784	European	ebi-a-GCST90018866	34 594 039	6
Membranous nephropathy	NIH	7979	European	ebi-a-GCST010005	32 231 244	4
Nephrotic syndrome	FinnGen	215 099	European	finn-b-N14_NEPHROTICSYND	NA	4
Calculus of kidney and ureter	FinnGen	218 414	European	finn-b-N14_CALCUKIDUR	NA	9
Microalbumin in urine	UKB	108 706	European	ukb-d-30500_irnt	NA	5
Urinary albumin excretion	NIH	382 500	European	ebi-a-GCST006586	30 220 432	32
Potassium in urine	MRC	395 995	European	ebi-a-GCST90013988	34 017 140	13
Creatinine (enzymatic) in urine	Neale Lab	327 525	European	ukb-a-333	NA	20
Sodium in urine	Neale Lab	326 831	European	ukb-a-335	NA	29
Serum creatinine (eGFRcrea)	CKDGen	133 814	European	ieu-a-1105	26 831 199	42
Serum cystatin C (eGFRcys)	CKDGen	33 152	European	ieu-a-1106	26 831 199	4
Kidney injury molecule 1 levels	CKDGen	21 758	European	ebi-a-GCST90012041	NA	12

Abbreviations: GWAS, genome-wide association study; IEU, Integrative Epidemiology Unit; IgA, immunoglobulin A; MRC, Medical Research Council; NA, not available; NIH, National Institutes of Health; PRACTICAL: Prostate Cancer Association Group to Investigate Cancer Associated Alterations in the Genome; SNP, single nucleotide polymorphism; UKB, UK Biobank.

### Causal effects of prostate diseases on ED

**Figure 2:**
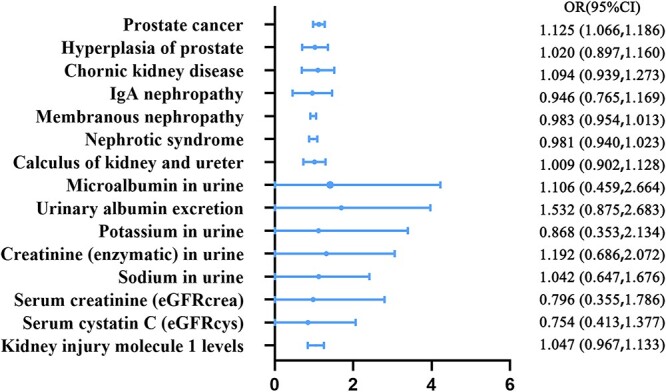
Causal links between prostate diseases, renal diseases, renal function and erectile dysfunction(inverse variance weighting Mendelian randomization randomization).

Following the completion of our MR analysis, we specifically identified a statistically significant association between PCa and an increased risk of ED. The association was found using the IVW method (odds ratio [OR], 1.125; 95% confidence interval [CI], 1.066-1.186; *P <* .0001) (Figure 2), and was supported by the weighted median method (OR, 1.117; 95% CI, 1.033-1.208; *P =* .006) and weighted mode method (OR, 1.119; 95% CI, 1.025-1.222; *P =* .013). No evidence of pleiotropy was observed in the MR-Egger regression (intercept = 0.009; *P =* .063). However, the presence of heterogeneity was detected in both the IVW (Q = 149.995; *P =* .021) and MR-Egger (Q = 145.575; *P =* .033) models, as supported by the findings presented in Table 2 and the funnel plot. Furthermore, our analysis conducted using leave-one-out methodology did not identify any influential SNPs, and the impact of each SNP on psoriasis risk is visualized in the forest plot presented in Supplementary Figure 1. However, the analysis presented in Supplementary Figure 2 indicates that there is no causal relationship between BPH and ED (*P* > .05).

**Table 2:** Heterogeneity tests and pleiotropy test for causality between exposure and erectile dysfunction.

**Exposure**	**Pleiotropy test**			**Heterogeneity test**					
				**Inverse variance weighted**			**MR-Egger**		
	egger_intercept	SE	*P*	Cochran’s Q	Q_df	*P*	Cochran’s Q	Q_df	*P*
Prostate cancer	0.009	0.005	.063	149.995	117	.021	145.575	116	.033
Hyperplasia of prostate	0.008	0.025	.758	12.320	9	.196	12.166	8	.144
Chronic kidney disease	0.011	0.038	.808	0.292	3	.961	0.216	2	.898
IgA nephropathy	0.025	0.038	.550	2.323	5	.803	1.897	4	.755
Membranous nephropathy	−0.015	0.026	.620	0.737	3	.865	0.399	2	.819
Nephrotic syndrome	0.043	0.062	.554	1.029	3	.794	0.533	2	.766
Calculus of kidney and ureter	−0.023	0.063	.723	8.815	8	.358	8.647	7	.279
Microalbumin in urine	−0.065	0.034	.147	8.606	4	.072	3.809	3	.283
Urinary albumin excretion	−0.004	0.011	.721	18.623	31	.961	18.493	30	.950
Potassium in urine	−0.069	0.047	.167	15.373	12	.222	12.822	11	.305
Creatinine (enzymatic) in urine	0.009	0.022	.706	11.897	19	.890	11.750	18	.860
Sodium in urine	0.007	0.017	.679	25.523	28	.599	25.348	27	.555
Serum creatinine (eGFRcrea)	0.005	0.012	.675	33.146	41	.804	32.967	40	.777
Serum cystatin C (eGFRcys)	−0.010	0.016	.592	0.504	3	.918	0.105	2	.949
Kidney injury molecule 1 levels	−0.018	0.011	.139	12.024	11	.362	9.446	10	.490

Abbreviation: df, degrees of freedom.

### Causal effects of renal diseases and renal function on ED

The analysis depicted in Figure 2 and Supplementary Figures 3 to 16 revealed no causal relationship between CKD, IgA nephropathy, membranous nephropathy, nephrotic syndrome, kidney and ureter calculi, or the investigated 8 final renal function parameters with ED (all *P* > .05). The MR-Egger intercept results (Table 2) indicated the absence of pleiotropy (*P* > .05), and the Cochran’s Q test and funnel plot provided no evidence of heterogeneity. Moreover, the robustness of the MR estimation was confirmed through leave-one-out analysis.

## Discussion

In our 2-sample MR analysis, we found evidence suggesting a potential correlation between PCa and an increased risk of ED. However, no significant associations were observed between BPH, CKD, IgA nephropathy, membranous nephropathy, nephrotic syndrome, kidney and ureter calculi, and the investigated renal function parameters.

In 1987, Mandel and Schuman^33^ conducted a case–control study to investigate the cross-sectional association between PCa and the risk of ED. Although the results did not reach statistical significance, Mandel and Schuman’s pioneering work laid the foundation for further research in this field. In 2011, Chung et al^34^ conducted a substantial survey, revealing that individuals with ED exhibited a 1.42-fold increase in the risk of developing cancer during a 5-year follow-up period compared with the control group, after adjusting for numerous demographic and sociological factors (95% CI, 1.03-2.09; *P =* .039). A recent study conducted among elderly veterans in the United States indicated a correlation between enhanced sexual function and a decreased overall risk of PCa.^35^ The current body of research primarily focuses on assessing the impact of erectile function as a precursor to PCa development, with a limited number of studies approaching the topic from the perspective of PCa as the point of origin. Our study serves to address critical gaps in existing research, presenting novel findings by establishing causal associations for the first time in this particular domain. Previous studies have found a link between ED and PCa risk, with common risk factors such as age, race, chronic disease, reduced ejaculation leading to the accumulation of harmful substances, and psychological factors being key contributors.^36^ The impact of inflammation and oxidative stress on the risk of ED in PCa patients is worth considering. PCa initiation and progression exhibit a notable correlation with chronic inflammation and infection.^37^ Additionally, infiltration of inflammatory cells, whether acute or chronic, triggers the augmentation of prostatic proliferative inflammatory atrophy—a state characterized by atrophic lesions within the prostate.^38^ In individuals with PCa, the prolonged inflammatory state and elevated levels of reactive oxygen species are associated with increased arterial stiffness, impaired arterial elasticity, and reduced blood flow necessary for penile erection.^9^ These conditions contribute to the development of vascular endothelial dysfunction, further diminishing the ability to achieve and maintain erections.

In a comprehensive study encompassing men across various stages of CKD, the prevalence of ED was observed to be 72.3%, 81.5%, and 85.7% in CKD stages 3, 4, and 5, respectively.^39^ This aligns with findings from 2 substantial population studies conducted in Brazil and China, which produced similar results. Costa et al^40^ demonstrated a prevalence of ED reaching 71.0% among CKD stages 4 and 5 patients 50 years of age and older, while Ye et al^41^ reported an ED prevalence of 80.6% among 176 peritoneal dialysis patients. Indeed, our study’s lack of positive findings does not dismiss the well-established high prevalence of ED among patients with CKD. Our findings do not support the conclusion that CKD is associated with ED. The complications that often accompany CKD may offer a partial explanation for the differing results obtained compared with previous studies. CKD frequently coexists with cardiovascular disease, with the latter commonly characterized by endothelial dysfunction and oxidative stress. The impaired production of nitric oxide in endothelial cells limits its beneficial effects, resulting in restricted smooth muscle dilation within the corpus cavernosum.^42^ Additionally, heightened levels of reactive oxygen species promote arterial stiffness, contributing to the progression of atherosclerosis and vascular endothelial dysfunction, ultimately leading to reduced blood flow to the penis.^43^ Moreover, it is significant to acknowledge that patients with CKD commonly experience lowered erythropoietin levels and exhibit heightened levels of prolactin, which ultimately leads to an increased incidence of anemia and decreased levels of hemoglobin. Previous studies have demonstrated that these factors can influence erectile function by impacting hormone secretion.^44^ Finally, psychological factors play a significant role in the development and progression of ED following CKD.

In our study, we found no significant associations between several key renal function measures and ED. This intriguing finding presents a contradiction to the conclusions drawn from previous cross-sectional observations. Notably, studies conducted in Japan and China have previously reported a higher likelihood of diabetic patients with macroalbuminuria or elevated urinary albumin-to-creatinine ratio achieving lower International Index of Erectile Function scores in comparison with normoalbuminuric patients.^45^^,^^46^ The decline in renal function and estimated eGFR have been implicated in the disruption of hormonal balance within the hypothalamic-pituitary-gonadal axis, resulting in reduced libido. Specifically, this hormonal imbalance is characterized by lowered levels of both total and free testosterone.^47^ Furthermore, as creatinine levels rise and eGFR declines, ED-related factors such as hyperparathyroidism, autonomic neuropathy, and vascular-related diseases are likely to advance.^48^ Hence, we propose that forthcoming cross-sectional or cohort studies should consider incorporating testosterone and free testosterone as potential confounding factors.

Interestingly, a recent meta-analysis conducted by Pyrgidis et al^49^ unveiled a noteworthy finding regarding the impact of kidney transplantation on erectile function. The study revealed a statistically significant improvement in erectile function among individuals who had undergone kidney transplantation (Relative Risk, 2.53; 95% CI, 1.44-4.44). This finding sheds new light on the potential benefits of kidney transplantation beyond its primary therapeutic goals. Drawing from their expertise, it can be deduced that kidney transplantation holds greater potential for restoring erectile function in patients. This effect is mediated through mechanisms such as enhanced hormone secretion, the cessation of dialysis dependence, and an overall improvement in quality of life, rather than by increasing the eGFR.^49^ A meta-analysis conducted by Kang et al^50^ revealed that renal transplant recipients experience an increase in serum testosterone levels as well as a decrease in prolactin and luteinizing hormone levels.

There are several limitations of this study. First, in our study, we specifically focused on prostate disease and kidney disease, incorporating and analyzing all related conditions. However, it is crucial to highlight that certain diseases, such as prostatitis, renal cell carcinoma, congenital kidney malformations, and others, lacked sufficient available SNPs in the existing published public data. As a result, we were unable to include these specific diseases in our study due to insufficient genetic information. Furthermore, conducting a bidirectional MR analysis is not currently feasible, and performing MR analysis with ED as an exposure factor still encounters challenges in terms of SNP availability. Therefore, it is common for the existing published MR studies to primarily utilize ED as an outcome instead of an exposure. Based on the present findings, our belief is that future research should primarily focus on bridging the gap in sample size and sample quality of SNPs in the ED population. Additionally, it is crucial to further investigate the pathophysiological mechanisms and key loci associated with the established link between PCa and ED. The association SNPs presented in this study (Supplementary Table 2) can serve as a foundation for forthcoming research in this area.

## Conclusion

The results of this study indicate a potential association between PCa and an elevated risk of ED. Further research is necessary to gain a better understanding and confirm these findings.

## Supplementary Material

Supplementary_Material_qfae002

## Data Availability

All data in the MR analyses are available from public databases (https://gwas.mrcieu.ac.uk/).
