# Supplementary material for: Causal associations between prostate diseases, renal diseases, renal function, and erectile dysfunction risk: a 2-sample Mendelian randomization study
Source: Sex Med. 2024 Feb 10;12(1):qfae002. doi: 10.1093/sexmed/qfae002 (PMC10859556; doi:10.1093/sexmed/qfae002)
Supplement: Supplementary_Material_qfae002 [file supplementary_material_qfae002.docx]

**Table1.** **Causal associations between prostate diseases, renal diseases, renal function and Erectile Dysfunction.**

| **Exposures** | **nSNP** | **OR** | **95%CI** | **P value** |
| --- | --- | --- | --- | --- |
| **Prostate cancer** |  |  |  |  |
| MR Egger | 118 | 1.033 | 0.931，1.145 | 0.541 |
| Weighted median | 118 | 1.117 | 1.033，1.208 | 0.006 |
| Inverse variance weighted | 118 | 1.125 | 1.066，1.186 | <0.0001 |
| Simple mode | 118 | 1.047 | 0.896，1.224 | 0.564 |
| Weighted mode | 118 | 1.119 | 1.025，1.222 | 0.013 |
| **Hyperplasia of prostate** |  |  |  |  |
| MR Egger | 10 | 0.965 | 0.670，1.391 | 0.855 |
| Weighted median | 10 | 0.984 | 0.842，1.150 | 0.836 |
| Inverse variance weighted | 10 | 1.020 | 0.897，1.160 | 0.761 |
| Simple mode | 10 | 0.903 | 0.684，1.192 | 0.489 |
| Weighted mode | 10 | 0.896 | 0.699，1.149 | 0.409 |
| **Chornic kidney disease** |  |  |  |  |
| MR Egger | 4 | 1.017 | 0.595，1.739 | 0.957 |
| Weighted median | 4 | 1.089 | 0.910，1.303 | 0.351 |
| Inverse variance weighted | 4 | 1.094 | 0.939，1.273 | 0.250 |
| Simple mode | 4 | 1.086 | 0.869，1.356 | 0.521 |
| Weighted mode | 4 | 1.085 | 0.879，1.339 | 0.503 |
| **IgA nephropathy** |  |  |  |  |
| MR Egger | 6 | 0.780 | 0.420，1.446 | 0.474 |
| Weighted median | 6 | 0.930 | 0.712，1.215 | 0.596 |
| Inverse variance weighted | 6 | 0.946 | 0.765，1.169 | 0.605 |
| Simple mode | 6 | 0.931 | 0.636，1.363 | 0.727 |
| Weighted mode | 6 | 0.886 | 0.594，1.321 | 0.578 |
| **Membranous nephropathy** |  |  |  |  |
| MR Egger | 4 | 1.004 | 0.929，1.085 | 0.927 |
| Weighted median | 4 | 0.979 | 0.946，1.013 | 0.225 |
| Inverse variance weighted | 4 | 0.983 | 0.954，1.013 | 0.275 |
| Simple mode | 4 | 0.969 | 0.924，1.017 | 0.298 |
| Weighted mode | 4 | 0.997 | 0.961，1.033 | 0.869 |
| **Nephrotic syndrome** |  |  |  |  |
| MR Egger | 4 | 0.955 | 0.877，1.040 | 0.401 |
| Weighted median | 4 | 0.980 | 0.936，1.027 | 0.398 |
| Inverse variance weighted | 4 | 0.981 | 0.940，1.023 | 0.370 |
| Simple mode | 4 | 0.990 | 0.935，1.048 | 0.747 |
| Weighted mode | 4 | 0.978 | 0.928，1.031 | 0.470 |
| **Calculus of kidney and ureter** |  |  |  |  |
| MR Egger | 9 | 1.182 | 0.505，2.765 | 0.711 |
| Weighted median | 9 | 1.004 | 0.868，1.160 | 0.961 |
| Inverse variance weighted | 9 | 1.009 | 0.902，1.128 | 0.881 |
| Simple mode | 9 | 0.870 | 0.681，1.112 | 0.299 |
| Weighted mode | 9 | 0.931 | 0.745，1.164 | 0.550 |
| **Microalbumin in urine** |  |  |  |  |
| MR Egger | 5 | 3.547 | 0.915，13.754 | 0.164 |
| Weighted median | 5 | 1.194 | 0.578，2.466 | 0.632 |
| Inverse variance weighted | 5 | 1.106 | 0.459，2.664 | 0.822 |
| Simple mode | 5 | 1.155 | 0.446，2.990 | 0.781 |
| Weighted mode | 5 | 1.155 | 0.521，2.562 | 0.740 |
| **Urinary albumin excretion** |  |  |  |  |
| MR Egger | 32 | 1.942 | 0.476，7.932 | 0.363 |
| Weighted median | 32 | 1.195 | 0.520，2.744 | 0.675 |
| Inverse variance weighted | 32 | 1.532 | 0.875，2.683 | 0.136 |
| Simple mode | 32 | 0.541 | 0.122，2.403 | 0.425 |
| Weighted mode | 32 | 1.578 | 0.483，5.160 | 0.456 |
| **Potassium in urine** |  |  |  |  |
| MR Egger | 13 | 70.286 | 0.196，25256.250 | 0.184 |
| Weighted median | 13 | 1.438 | 0.473，4.369 | 0.522 |
| Inverse variance weighted | 13 | 0.868 | 0.353，2.134 | 0.758 |
| Simple mode | 13 | 1.506 | 0.285，7.952 | 0.638 |
| Weighted mode | 13 | 1.783 | 0.350，9.096 | 0.500 |
| **Creatinine (enzymatic) in urine** |  |  |  |  |
| MR Egger | 20 | 0.782 | 0.085，7.236 | 0.831 |
| Weighted median | 20 | 1.463 | 0.682，3.142 | 0.329 |
| Inverse variance weighted | 20 | 1.192 | 0.686，2.072 | 0.534 |
| Simple mode | 20 | 1.553 | 0.402，5.993 | 0.531 |
| Weighted mode | 20 | 0.852 | 0.236，3.074 | 0.810 |
| **Sodium in urine** |  |  |  |  |
| MR Egger | 29 | 0.702 | 0.104，4.750 | 0.719 |
| Weighted median | 29 | 1.106 | 0.569，2.150 | 0.767 |
| Inverse variance weighted | 29 | 1.042 | 0.647，1.676 | 0.866 |
| Simple mode | 29 | 1.974 | 0.404，9.658 | 0.408 |
| Weighted mode | 29 | 2.008 | 0.461，8.745 | 0.361 |
| **Serum creatinine (eGFRcrea)** |  |  |  |  |
| MR Egger | 42 | 0.443 | 0.026，7.535 | 0.576 |
| Weighted median | 42 | 0.567 | 0.179，1.789 | 0.333 |
| Inverse variance weighted | 42 | 0.796 | 0.355，1.786 | 0.580 |
| Simple mode | 42 | 0.329 | 0.040，2.713 | 0.308 |
| Weighted mode | 42 | 0.434 | 0.079，2.392 | 0.344 |
| **Serum cystatin C (eGFRcys)** |  |  |  |  |
| MR Egger | 4 | 0.938 | 0.379，2.323 | 0.903 |
| Weighted median | 4 | 0.781 | 0.419，1.458 | 0.438 |
| Inverse variance weighted | 4 | 0.754 | 0.413，1.377 | 0.358 |
| Simple mode | 4 | 0.421 | 0.104，1.699 | 0.311 |
| Weighted mode | 4 | 0.809 | 0.428，1.527 | 0.559 |
| **Kidney injury molecule 1 levels** |  |  |  |  |
| MR Egger | 12 | 1.113 | 1.000，1.238 | 0.078 |
| Weighted median | 12 | 1.050 | 0.959，1.150 | 0.288 |
| Inverse variance weighted | 12 | 1.047 | 0.967，1.133 | 0.260 |
| Simple mode | 12 | 1.049 | 0.865，1.270 | 0.638 |
| Weighted mode | 12 | 1.049 | 0.965，1.140 | 0.289 |

**TABLE 2 SNPs used as instrumental variables of PCa on ED in the MR analyses.**

| **SNP** | **Chr** | **Pos** | **Beta** | **SE** | **Effect_allele** | **Other_allele** | **EAF** | **F-statistic** | **P** |
| --- | --- | --- | --- | --- | --- | --- | --- | --- | --- |
| rs10127983 | 1 | 153923276 | 0.0631 | 0.0085 | T | C | 0.3117 | 55.10800308 | 1.02E-13 |
| rs12139208 | 1 | 88213014 | -0.0458 | 0.0082 | C | T | 0.6286 | 31.19586686 | 2.58E-08 |
| rs17599629 | 1 | 150658287 | 0.0654 | 0.0096 | G | A | 0.2181 | 46.40949445 | 9.87E-12 |
| rs4245739 | 1 | 204518842 | 0.0924 | 0.0091 | A | C | 0.7379 | 103.0991215 | 3.17E-24 |
| rs56103503 | 1 | 154980351 | 0.0652 | 0.0082 | T | C | 0.3876 | 63.2209902 | 1.81E-15 |
| rs7522437 | 1 | 204029890 | -0.0457 | 0.008 | A | G | 0.4609 | 32.63219091 | 1.21E-08 |
| rs823121 | 1 | 205724302 | -0.0472 | 0.008 | A | G | 0.4322 | 34.80950361 | 3.38E-09 |
| rs11691517 | 2 | 111893096 | -0.0635 | 0.0091 | G | T | 0.2589 | 48.69209637 | 3.51E-12 |
| rs1990613 | 2 | 10781975 | -0.0676 | 0.0079 | C | T | 0.495 | 73.22055497 | 1.59E-17 |
| rs2028900 | 2 | 85767735 | -0.082 | 0.008 | T | C | 0.4418 | 105.0610018 | 6.67E-25 |
| rs28485589 | 2 | 173303031 | -0.2441 | 0.0176 | G | A | 0.0608 | 192.3552438 | 1.11E-43 |
| rs34925593 | 2 | 174234547 | 0.0466 | 0.0084 | C | T | 0.4817 | 30.77563824 | 2.82E-08 |
| rs59015435 | 2 | 10129252 | 0.0915 | 0.0152 | A | G | 0.0701 | 36.23671491 | 1.98E-09 |
| rs62186433 | 2 | 242257835 | 0.0863 | 0.011 | G | A | 0.1486 | 61.55027931 | 3.21E-15 |
| rs6754084 | 2 | 202124997 | 0.05 | 0.0091 | C | T | 0.7324 | 30.18916013 | 3.53E-08 |
| rs7580494 | 2 | 62781499 | 0.0551 | 0.0081 | T | C | 0.3878 | 46.27292649 | 1.17E-11 |
| rs7591218 | 2 | 43637998 | -0.084 | 0.0085 | G | A | 0.6844 | 97.65950702 | 2.96E-23 |
| rs77482050 | 2 | 242139600 | -0.3939 | 0.0403 | A | G | 0.0145 | 95.53349721 | 1.51E-22 |
| rs77559646 | 2 | 242135265 | 0.2492 | 0.0267 | A | G | 0.0237 | 87.10986892 | 9.93E-21 |
| rs9306894 | 2 | 20878105 | 0.0777 | 0.0082 | G | A | 0.3633 | 89.7858999 | 1.92E-21 |
| rs12496052 | 3 | 127859075 | 0.083 | 0.0081 | T | G | 0.4218 | 104.9977406 | 2.29E-24 |
| rs12629813 | 3 | 113284149 | -0.0851 | 0.008 | T | C | 0.4371 | 113.1547927 | 1.98E-26 |
| rs1283104 | 3 | 106962521 | 0.047 | 0.0082 | G | C | 0.3788 | 32.8520003 | 8.81E-09 |
| rs75358080 | 3 | 87398945 | -0.1452 | 0.0216 | G | A | 0.0373 | 45.18762723 | 1.71E-11 |
| rs75550450 | 3 | 152043342 | -0.0885 | 0.0135 | T | A | 0.1031 | 42.97469582 | 5.59E-11 |
| rs78416326 | 3 | 170074517 | -0.1763 | 0.0102 | C | G | 0.2062 | 298.7432409 | 5.60E-67 |
| rs10007915 | 4 | 106065308 | -0.1202 | 0.0081 | G | C | 0.4093 | 220.2078033 | 8.27E-50 |
| rs12510147 | 4 | 95521863 | -0.0888 | 0.0086 | A | G | 0.3115 | 106.6161108 | 4.34E-25 |
| rs17804499 | 4 | 74442349 | -0.1542 | 0.0192 | C | G | 0.0534 | 64.50005679 | 9.15E-16 |
| rs10793821 | 5 | 133836209 | 0.0527 | 0.008 | T | C | 0.5795 | 43.39453744 | 5.43E-11 |
| rs1482680 | 5 | 44392142 | -0.0501 | 0.0085 | A | G | 0.6667 | 34.74012744 | 3.58E-09 |
| rs4976790 | 5 | 177968915 | 0.0737 | 0.0127 | T | G | 0.1131 | 33.67606513 | 6.73E-09 |
| rs9686557 | 5 | 172959030 | 0.0482 | 0.008 | C | A | 0.444 | 36.30010736 | 1.94E-09 |
| rs1048019 | 6 | 160147630 | -0.0569 | 0.0091 | G | A | 0.2849 | 39.09629069 | 3.22E-10 |
| rs10947980 | 6 | 41525739 | 0.0834 | 0.0089 | G | A | 0.2634 | 87.81038776 | 8.78E-21 |
| rs12665339 | 6 | 30601232 | 0.0615 | 0.0106 | G | A | 0.1674 | 33.66141034 | 5.56E-09 |
| rs2018336 | 6 | 11217897 | -0.0679 | 0.0096 | C | T | 0.2206 | 50.02543681 | 1.91E-12 |
| rs339351 | 6 | 117200434 | -0.0843 | 0.0087 | A | C | 0.3052 | 93.88807851 | 2.90E-22 |
| rs4711748 | 6 | 43694598 | -0.0521 | 0.0094 | C | T | 0.7746 | 30.71945782 | 3.36E-08 |
| rs6557265 | 6 | 153433402 | -0.077 | 0.008 | C | T | 0.4226 | 92.63930396 | 1.04E-21 |
| rs6941125 | 6 | 109287209 | 0.0704 | 0.0111 | A | T | 0.1457 | 40.22473278 | 1.99E-10 |
| rs9443189 | 6 | 76495882 | -0.0635 | 0.0116 | G | A | 0.1429 | 29.96575877 | 4.68E-08 |
| rs10486567 | 7 | 27976563 | -0.1335 | 0.0094 | A | G | 0.2373 | 201.6975538 | 2.04E-45 |
| rs17544073 | 7 | 47451572 | 0.0554 | 0.008 | T | C | 0.4394 | 47.95494116 | 3.36E-12 |
| rs17621345 | 7 | 40875192 | -0.0715 | 0.0095 | C | A | 0.2587 | 56.64462161 | 6.72E-14 |
| rs35355140 | 7 | 27204732 | -0.0854 | 0.0155 | A | C | 0.0781 | 30.35611239 | 3.33E-08 |
| rs4727386 | 7 | 97688440 | 0.1055 | 0.008 | A | G | 0.4604 | 173.9076763 | 1.17E-39 |
| rs917811 | 7 | 21059022 | -0.0686 | 0.0085 | T | C | 0.6856 | 65.13346566 | 5.51E-16 |
| rs11135766 | 8 | 23533623 | -0.1291 | 0.008 | T | C | 0.5736 | 260.4151927 | 5.09E-59 |
| rs11135910 | 8 | 25892142 | 0.0782 | 0.011 | T | C | 0.1529 | 50.53845287 | 9.19E-13 |
| rs11986220 | 8 | 128531689 | -0.355 | 0.0122 | T | A | 0.8953 | 846.7025188 | 1.10E-187 |
| rs2928681 | 8 | 23437981 | -0.0553 | 0.008 | A | C | 0.5887 | 47.78197488 | 5.38E-12 |
| rs4871743 | 8 | 127856177 | 0.0609 | 0.0096 | T | C | 0.7825 | 40.2425902 | 2.69E-10 |
| rs6983267 | 8 | 128413305 | -0.2004 | 0.0079 | T | G | 0.4892 | 643.4800084 | 2.81E-141 |
| rs72725868 | 8 | 128091418 | -0.1175 | 0.015 | G | A | 0.0881 | 61.36023611 | 5.46E-15 |
| rs73351629 | 8 | 128018466 | -0.1375 | 0.0085 | G | C | 0.3334 | 261.6744692 | 5.15E-59 |
| rs77541621 | 8 | 128077146 | 0.5907 | 0.0227 | A | G | 0.0243 | 677.1362036 | 1.03E-149 |
| rs10122495 | 9 | 34049779 | -0.0501 | 0.0088 | A | T | 0.704 | 32.41185702 | 1.34E-08 |
| rs1048169 | 9 | 19055965 | 0.0609 | 0.0081 | C | T | 0.379 | 56.52731463 | 6.53E-14 |
| rs1182 | 9 | 132576060 | 0.0581 | 0.0095 | A | C | 0.2198 | 37.40234753 | 1.10E-09 |
| rs12004058 | 9 | 18564470 | -0.0481 | 0.0086 | G | C | 0.312 | 31.28146306 | 2.36E-08 |
| rs77334358 | 9 | 110256979 | -0.2803 | 0.0358 | T | C | 0.0173 | 61.30190196 | 5.17E-15 |
| rs817833 | 9 | 110152977 | -0.0733 | 0.0131 | T | G | 0.8914 | 31.30827681 | 2.14E-08 |
| rs1004934 | 10 | 122796182 | 0.0572 | 0.0081 | C | G | 0.3874 | 49.86729682 | 1.39E-12 |
| rs12414066 | 10 | 45772665 | -0.0636 | 0.0106 | C | T | 0.1709 | 35.99948665 | 2.07E-09 |
| rs1935581 | 10 | 90195149 | -0.0477 | 0.0082 | T | C | 0.3728 | 33.83785774 | 6.55E-09 |
| rs34487581 | 10 | 897201 | 0.0775 | 0.0109 | A | G | 0.1585 | 50.55268371 | 9.34E-13 |
| rs4962419 | 10 | 126697114 | 0.0598 | 0.0089 | A | G | 0.2664 | 45.14567613 | 1.62E-11 |
| rs10840603 | 11 | 2233797 | -0.1705 | 0.0098 | G | A | 0.8014 | 302.6846675 | 4.38E-68 |
| rs11214775 | 11 | 113807181 | -0.071 | 0.009 | A | G | 0.2915 | 62.23368045 | 3.93E-15 |
| rs12285347 | 11 | 102396607 | -0.0745 | 0.0082 | C | T | 0.4507 | 82.54269563 | 1.17E-19 |
| rs12419854 | 11 | 68886460 | 0.0608 | 0.0108 | T | A | 0.1622 | 31.69227783 | 1.86E-08 |
| rs12795301 | 11 | 68992285 | 0.2201 | 0.0105 | A | C | 0.1586 | 439.3951855 | 3.07E-98 |
| rs17749618 | 11 | 76251818 | -0.0609 | 0.0085 | G | A | 0.324 | 51.33227838 | 7.48E-13 |
| rs1800057 | 11 | 108143456 | 0.15 | 0.026 | G | C | 0.0234 | 33.28354904 | 8.15E-09 |
| rs1881502 | 11 | 1507512 | -0.0581 | 0.0101 | C | T | 0.8097 | 33.0904996 | 7.42E-09 |
| rs375314312 | 11 | 47421965 | -0.0468 | 0.0085 | T | G | 0.5321 | 30.3143082 | 3.37E-08 |
| rs878987 | 11 | 134266372 | 0.0639 | 0.0117 | G | A | 0.1459 | 29.82797702 | 4.77E-08 |
| rs10774740 | 12 | 114666202 | -0.0741 | 0.0082 | T | G | 0.3844 | 81.65871062 | 1.63E-19 |
| rs10845938 | 12 | 14416918 | 0.0572 | 0.008 | G | A | 0.5541 | 51.121771 | 9.80E-13 |
| rs35644221 | 12 | 90227779 | -0.0604 | 0.0088 | A | G | 0.3192 | 47.10883236 | 8.14E-12 |
| rs61455481 | 12 | 49701111 | 0.0564 | 0.01 | A | G | 0.195 | 31.8091464 | 1.46E-08 |
| rs7295014 | 12 | 133067989 | -0.0516 | 0.0084 | A | G | 0.6578 | 37.73415579 | 9.50E-10 |
| rs73110464 | 12 | 53312612 | 0.1583 | 0.0116 | T | C | 0.1277 | 186.2257184 | 1.11E-42 |
| rs7968403 | 12 | 65012824 | -0.0589 | 0.0085 | C | T | 0.3575 | 48.01606269 | 3.38E-12 |
| rs80130819 | 12 | 48419618 | -0.0957 | 0.0142 | C | A | 0.0916 | 45.41935827 | 1.89E-11 |
| rs7996468 | 13 | 73714290 | -0.0685 | 0.0099 | T | C | 0.7756 | 47.87453412 | 4.37E-12 |
| rs1004030 | 14 | 23305649 | -0.0462 | 0.0082 | C | T | 0.4156 | 31.74315234 | 1.55E-08 |
| rs11629412 | 14 | 37138294 | 0.0573 | 0.0082 | C | G | 0.5821 | 48.82872071 | 2.34E-12 |
| rs62003551 | 14 | 53424320 | -0.1005 | 0.0117 | G | A | 0.1425 | 73.78264279 | 8.46E-18 |
| rs767127 | 14 | 69134264 | 0.0512 | 0.0079 | G | A | 0.4989 | 42.00292611 | 1.00E-10 |
| rs33984059 | 15 | 56385868 | -0.1761 | 0.0308 | G | A | 0.0225 | 32.68971136 | 1.10E-08 |
| rs4924487 | 15 | 40922915 | 0.0622 | 0.0109 | C | G | 0.8359 | 32.5627879 | 1.32E-08 |
| rs80326387 | 15 | 66705043 | 0.0567 | 0.0091 | A | G | 0.2583 | 38.8219316 | 4.64E-10 |
| rs11863709 | 16 | 57654576 | -0.1481 | 0.022 | T | C | 0.0403 | 45.31672981 | 1.78E-11 |
| rs13332673 | 16 | 89940386 | 0.2246 | 0.0394 | T | G | 0.0129 | 32.49532367 | 1.18E-08 |
| rs16958674 | 16 | 57693055 | -0.0533 | 0.0094 | T | C | 0.2385 | 32.15085434 | 1.27E-08 |
| rs8052913 | 16 | 82166181 | -0.0458 | 0.0081 | T | C | 0.6159 | 31.97088993 | 1.71E-08 |
| rs12944450 | 17 | 46546346 | 0.1552 | 0.0237 | T | C | 0.0372 | 42.88254468 | 6.26E-11 |
| rs28441558 | 17 | 7803118 | 0.1507 | 0.0182 | C | T | 0.0548 | 68.56106192 | 1.02E-16 |
| rs3110641 | 17 | 36047417 | -0.0691 | 0.0095 | G | A | 0.778 | 52.90572756 | 2.62E-13 |
| rs684232 | 17 | 618965 | 0.0832 | 0.0082 | C | T | 0.3534 | 102.9467771 | 4.34E-24 |
| rs9907478 | 17 | 47395729 | 0.1064 | 0.0147 | T | C | 0.0757 | 52.3892756 | 4.52E-13 |
| rs9911515 | 17 | 69115358 | -0.1618 | 0.0079 | G | A | 0.5197 | 419.4658979 | 6.47E-93 |
| rs10460109 | 18 | 73036165 | -0.0441 | 0.008 | C | T | 0.5857 | 30.38722293 | 3.48E-08 |
| rs12956892 | 18 | 56746315 | 0.0498 | 0.0086 | T | G | 0.3021 | 33.53170139 | 7.68E-09 |
| rs71279357 | 18 | 76777252 | -0.0893 | 0.0093 | T | A | 0.2681 | 92.19998017 | 4.64E-22 |
| rs12610267 | 19 | 38744733 | -0.0966 | 0.0079 | G | A | 0.5103 | 149.5181371 | 4.87E-34 |
| rs2191139 | 19 | 42001210 | 0.0875 | 0.0093 | C | T | 0.7474 | 88.52053212 | 3.25E-21 |
| rs62113212 | 19 | 51360840 | -0.3028 | 0.0159 | T | C | 0.0775 | 362.6697225 | 4.26E-81 |
| rs1058319 | 20 | 62374389 | -0.1261 | 0.0124 | T | C | 0.1367 | 103.4143031 | 1.92E-24 |
| rs35477237 | 20 | 52455590 | -0.0682 | 0.0083 | G | A | 0.5153 | 67.51594824 | 2.74E-16 |
| rs61735792 | 21 | 42866332 | 0.23 | 0.0332 | A | G | 0.0158 | 47.99249315 | 4.50E-12 |
| rs9978557 | 21 | 42882462 | -0.1009 | 0.0143 | T | C | 0.098 | 49.78563658 | 2.02E-12 |
| rs1978060 | 22 | 19749525 | 0.0568 | 0.0083 | G | A | 0.6127 | 46.83109296 | 8.54E-12 |
| rs2007886 | 22 | 43502152 | -0.0979 | 0.0135 | C | T | 0.1065 | 52.58860537 | 4.58E-13 |
| rs34419824 | 22 | 40499103 | 0.0721 | 0.0099 | T | A | 0.1926 | 53.03883146 | 3.83E-13 |
| rs9625483 | 22 | 28888939 | 0.1338 | 0.024 | A | G | 0.0288 | 31.0801818 | 2.43E-08 |

SNP, single-nucleotide polymorphism; SE, standard error; OR, odds ratio; 95%CI, 95% confidence interval.


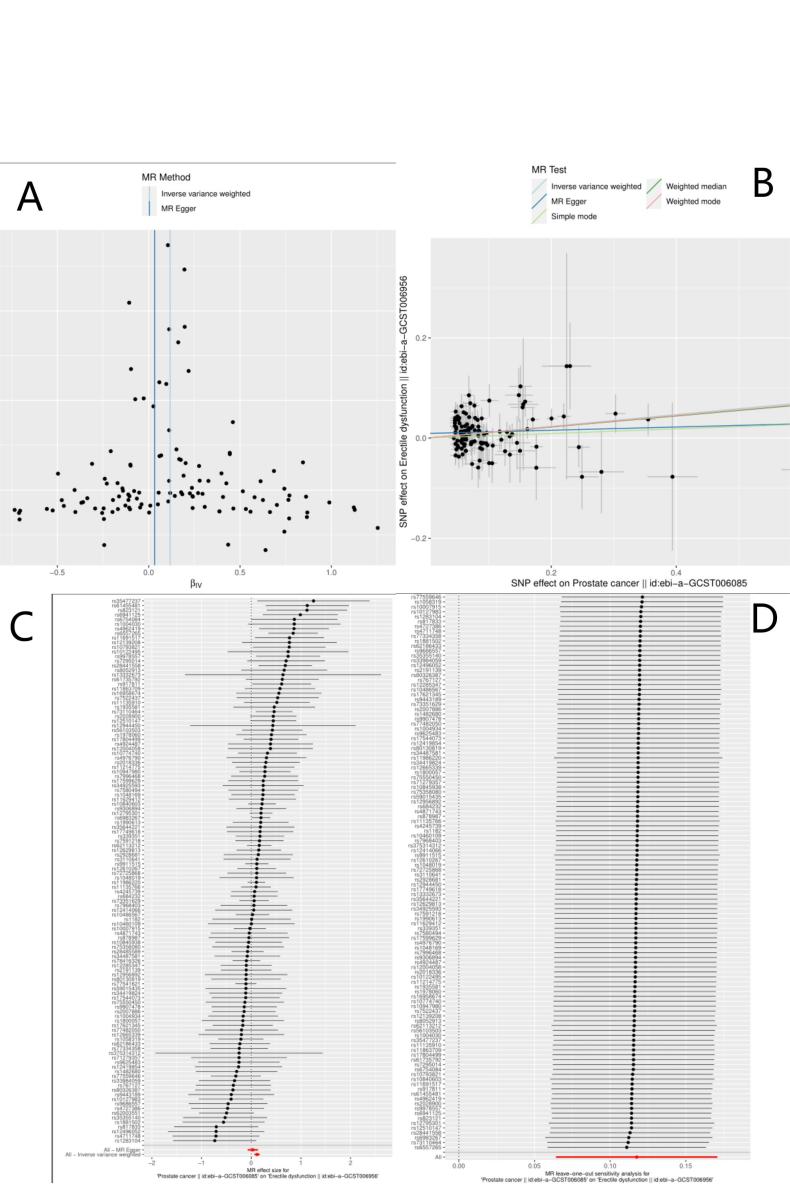


**Figure. 1 (A)Funnel plot; (B)Scatter plot; (C)Forest plot; (D)Sensitivity analysis of the effect of prostate cancer on erectile dysfunction.**

**
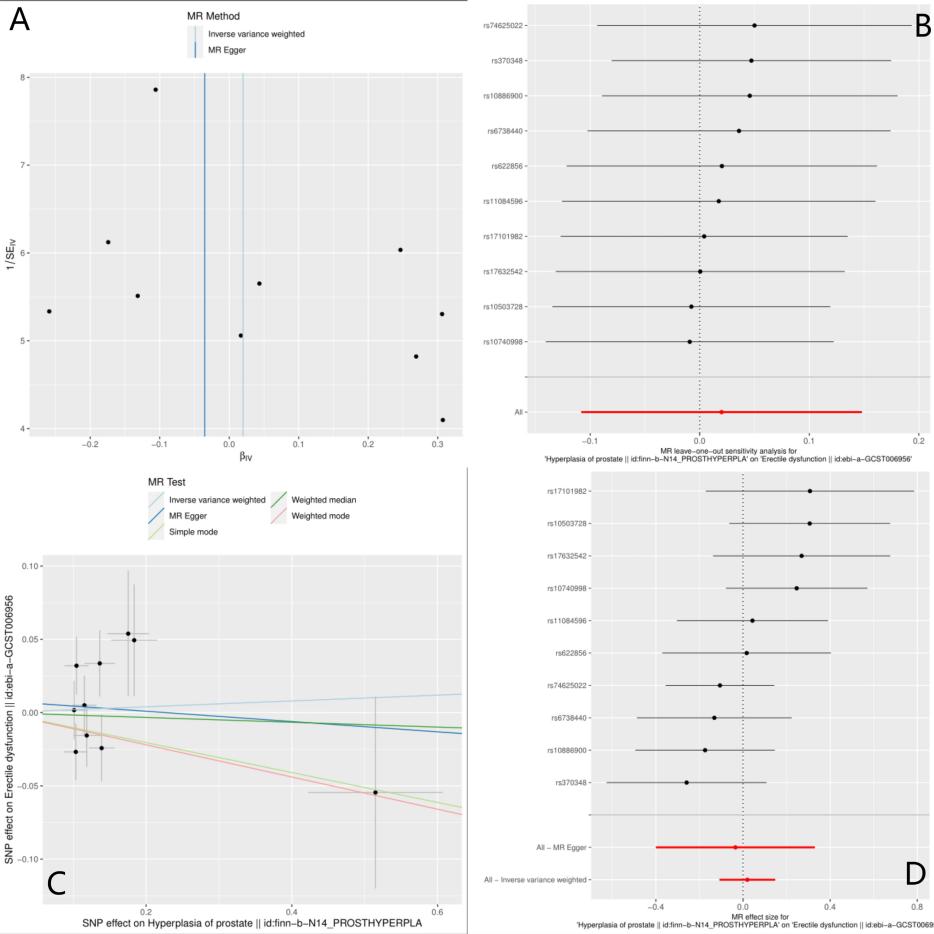
**

**Figure. 2 (A)Funnel plot; (B)Sensitivity analysis; (C)Scatter plot; (D)Forest plot of the effect of Hyperplasia of prostate on erectile dysfunction.**


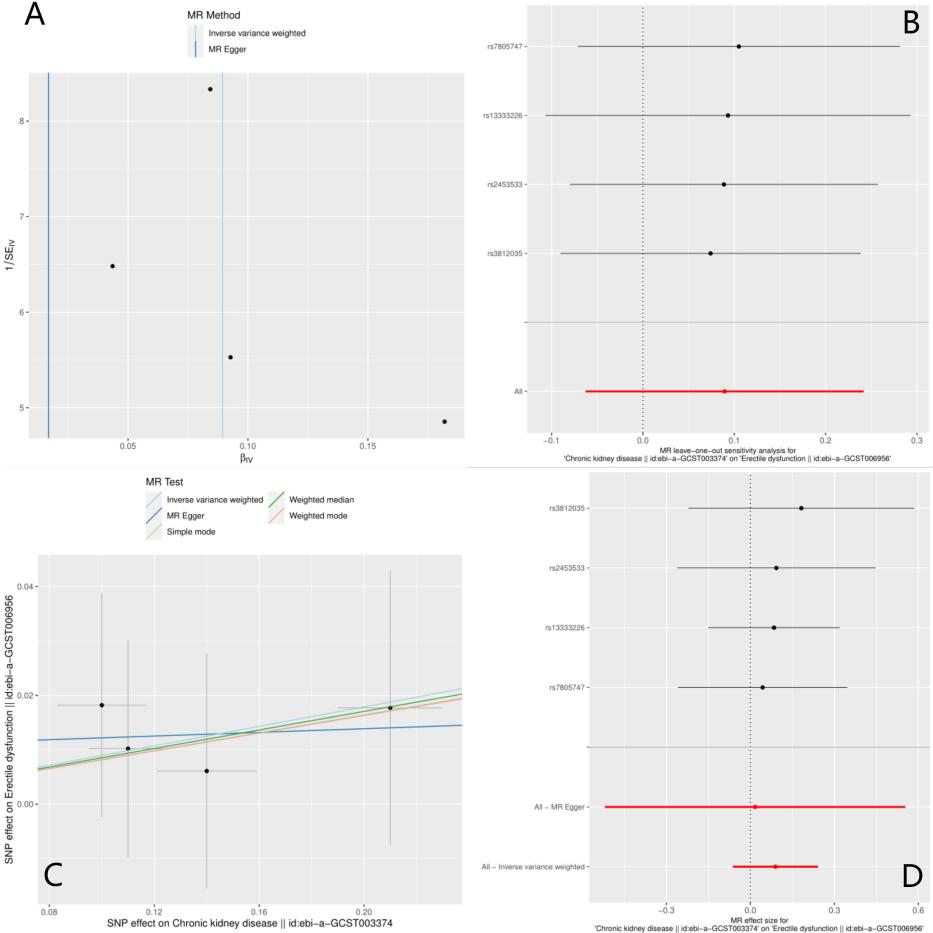


**Figure. 3 (A)Funnel plot; (B)Sensitivity analysis; (C)Scatter plot; (D)Forest plot of the effect of chronic kidney disease on erectile dysfunction.**


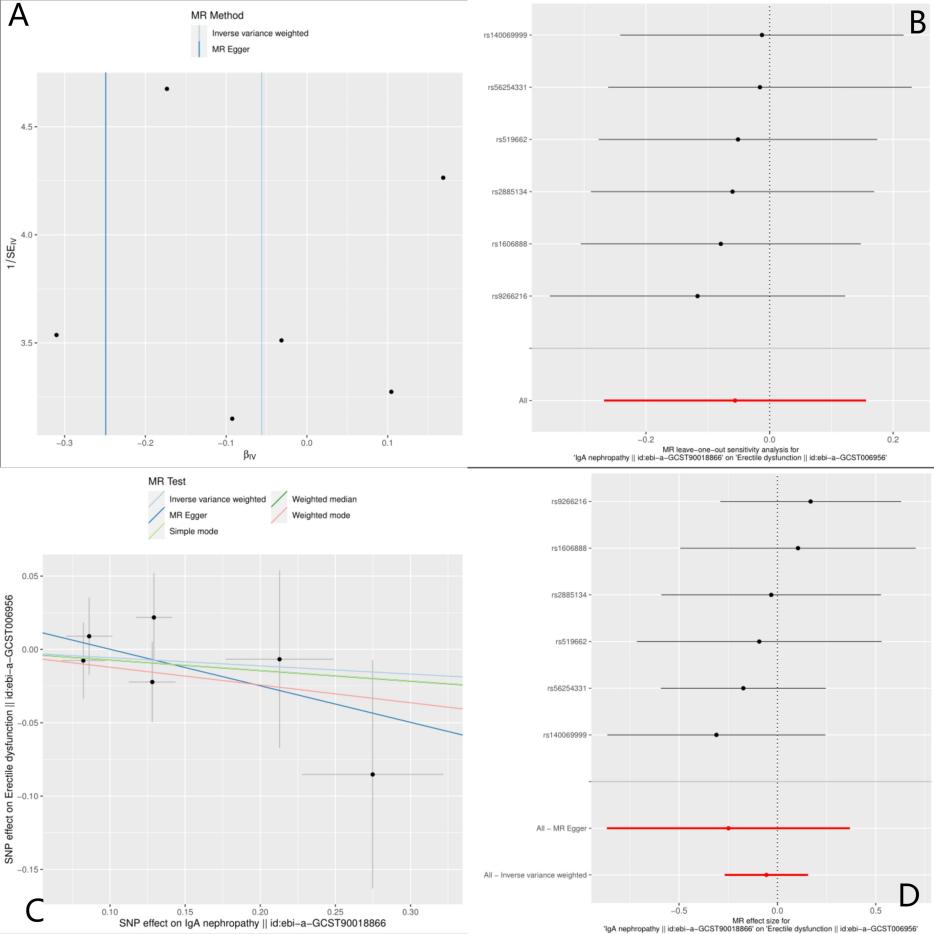


**Figure. 4 (A)Funnel plot; (B)Sensitivity analysis; (C)Scatter plot; (D)Forest plot of the effect of IgA nephropathy on erectile dysfunction.**


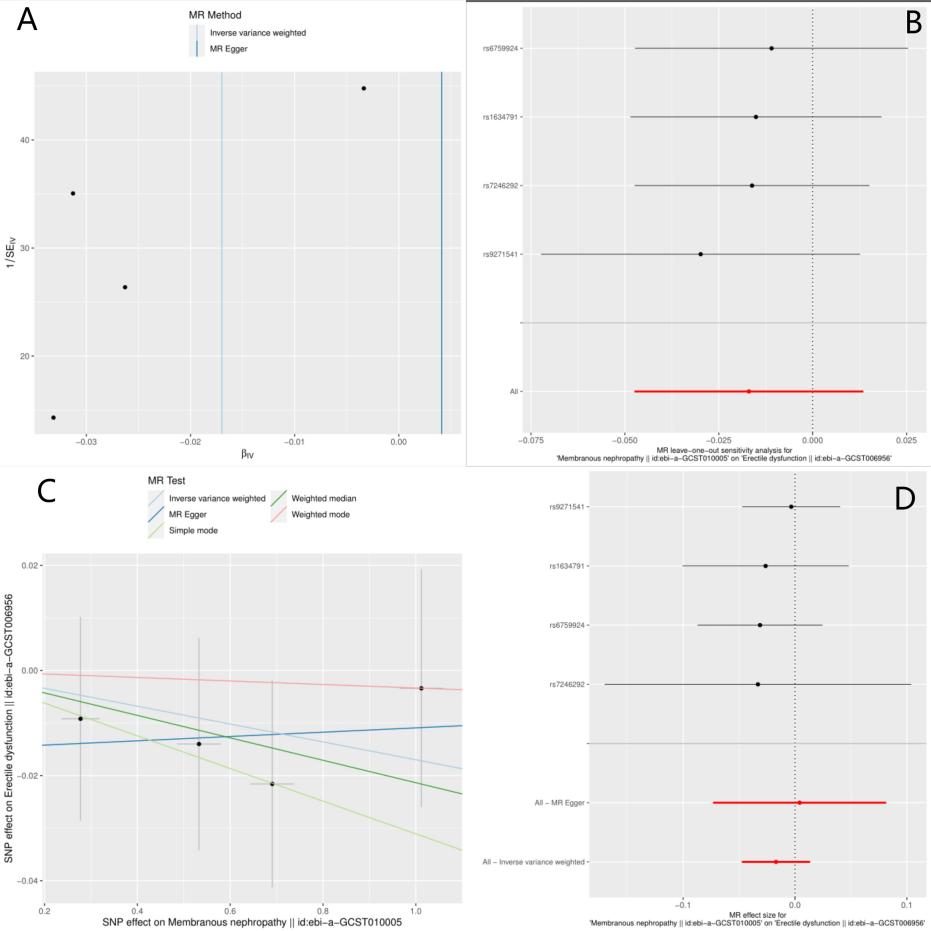


**Figure. 5 (A)Funnel plot; (B)Sensitivity analysis; (C)Scatter plot; (D)Forest plot of the effect of Membranous nephropathy on erectile dysfunction.**


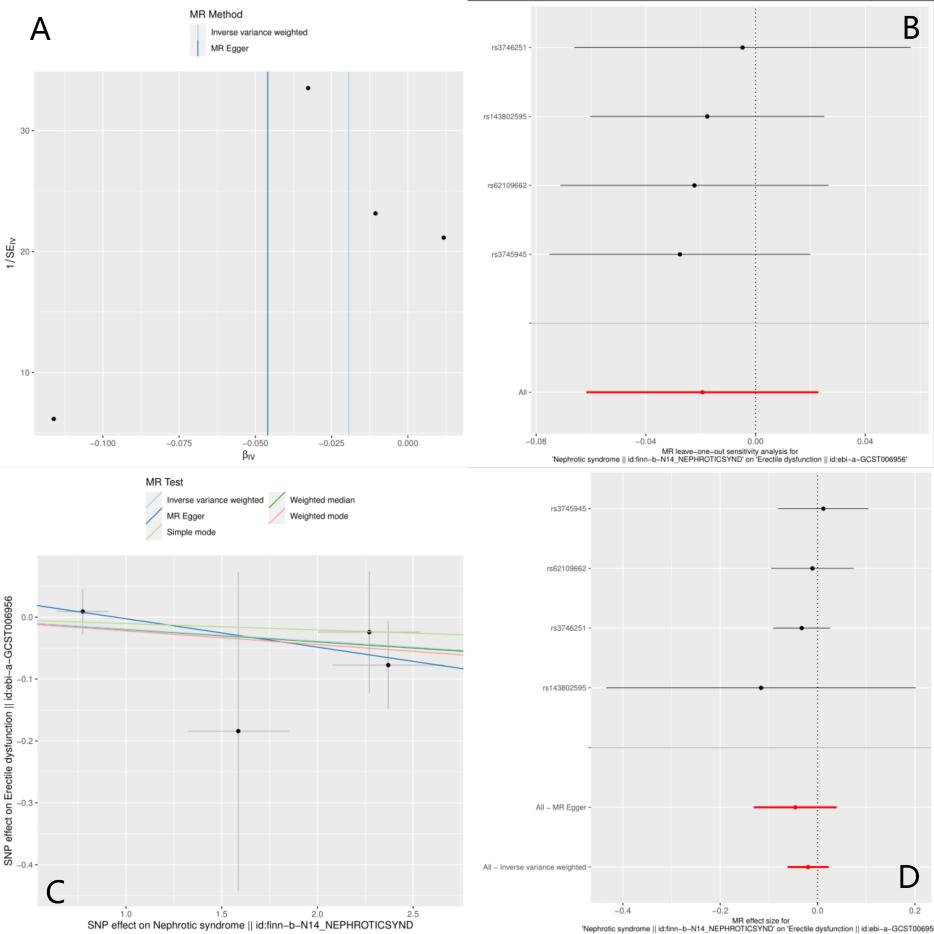


**Figure. 6 (A)Funnel plot; (B)Sensitivity analysis; (C)Scatter plot; (D)Forest plot of the effect of Nephrotic syndrome on erectile dysfunction.**


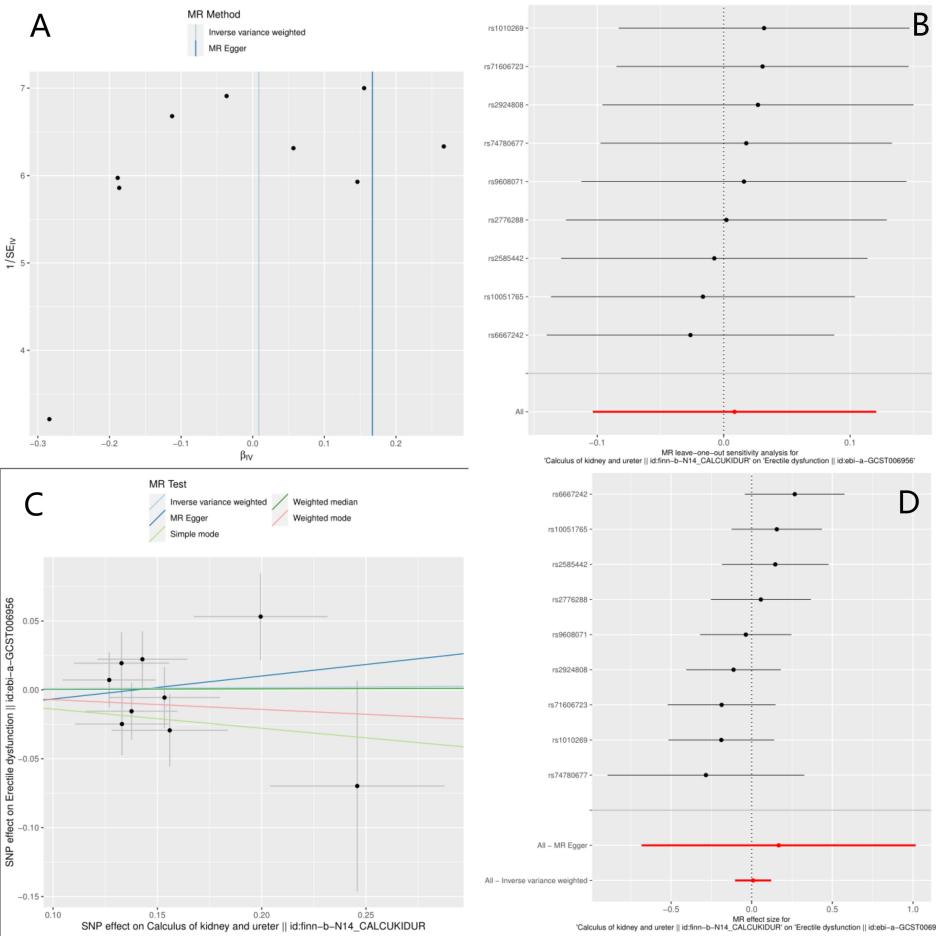


**Figure. 7 (A)Funnel plot; (B)Sensitivity analysis; (C)Scatter plot; (D)Forest plot of the effect of Calculus of kidney and ureter on erectile dysfunction.**


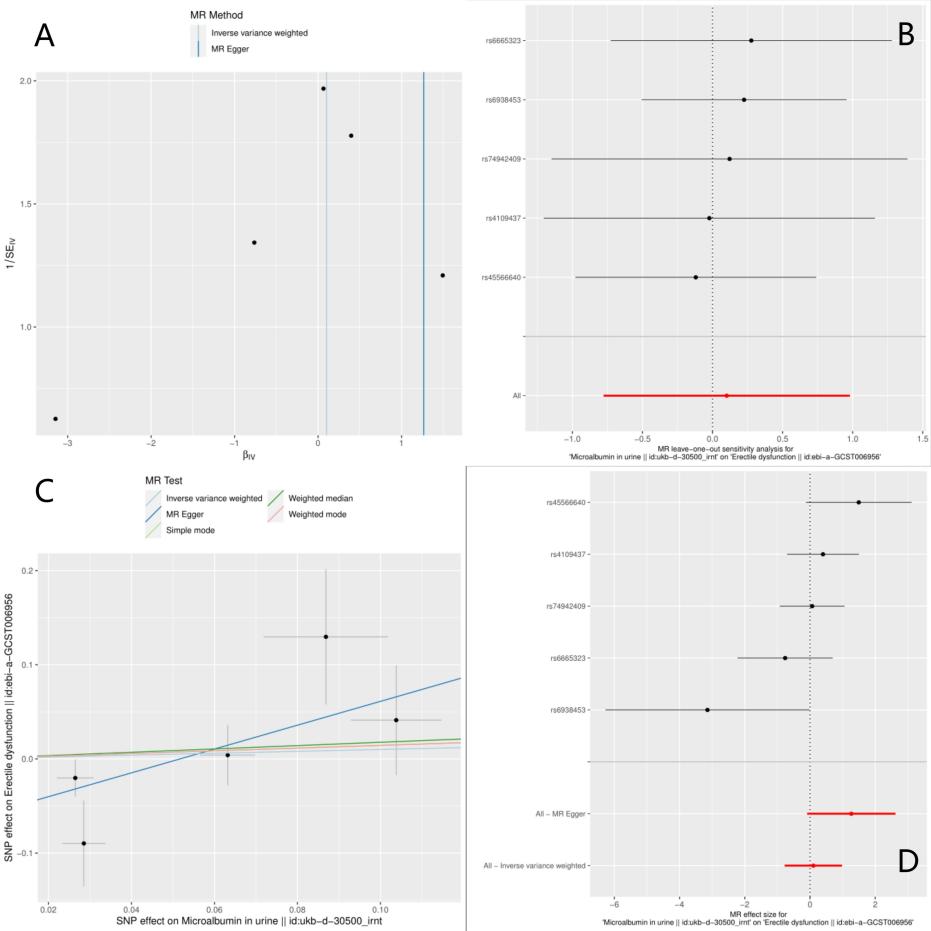


**Figure. 8 (A)Funnel plot; (B)Sensitivity analysis; (C)Scatter plot; (D)Forest plot of the effect of Microalbumin in urine on erectile dysfunction.**


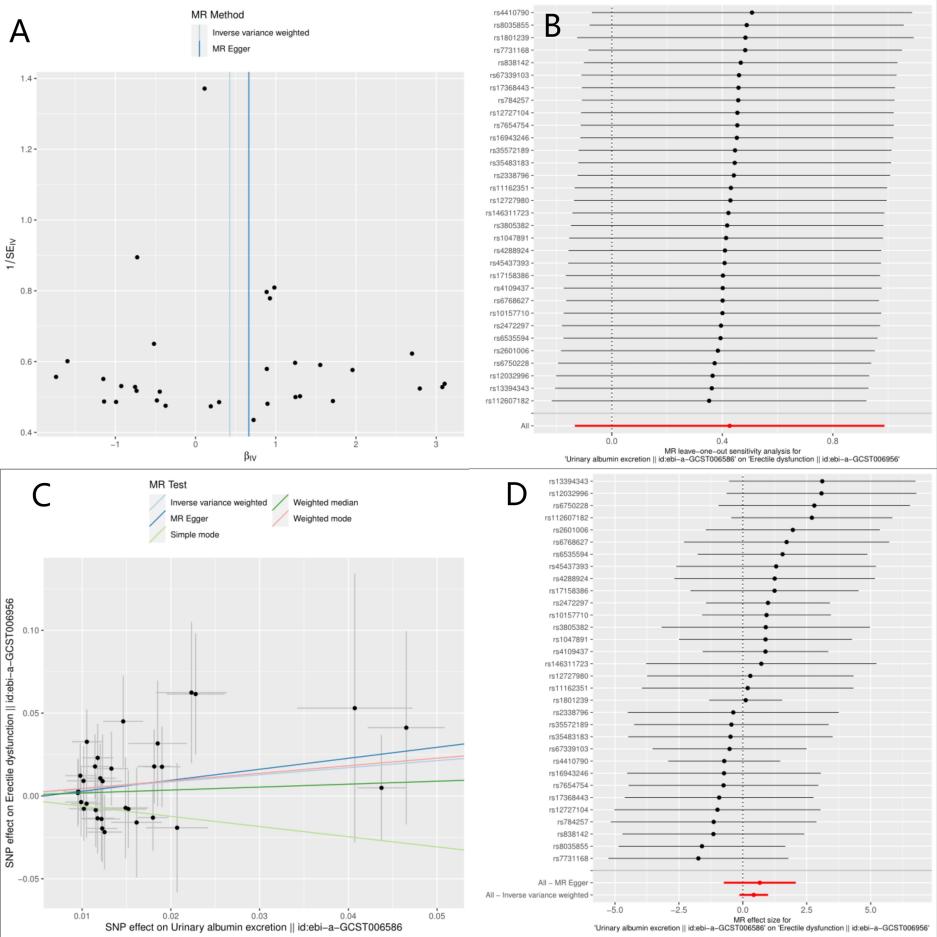


**Figure. 9 (A)Funnel plot; (B)Sensitivity analysis; (C)Scatter plot; (D)Forest plot of the effect of Urinary albumin excretion on erectile dysfunction.**


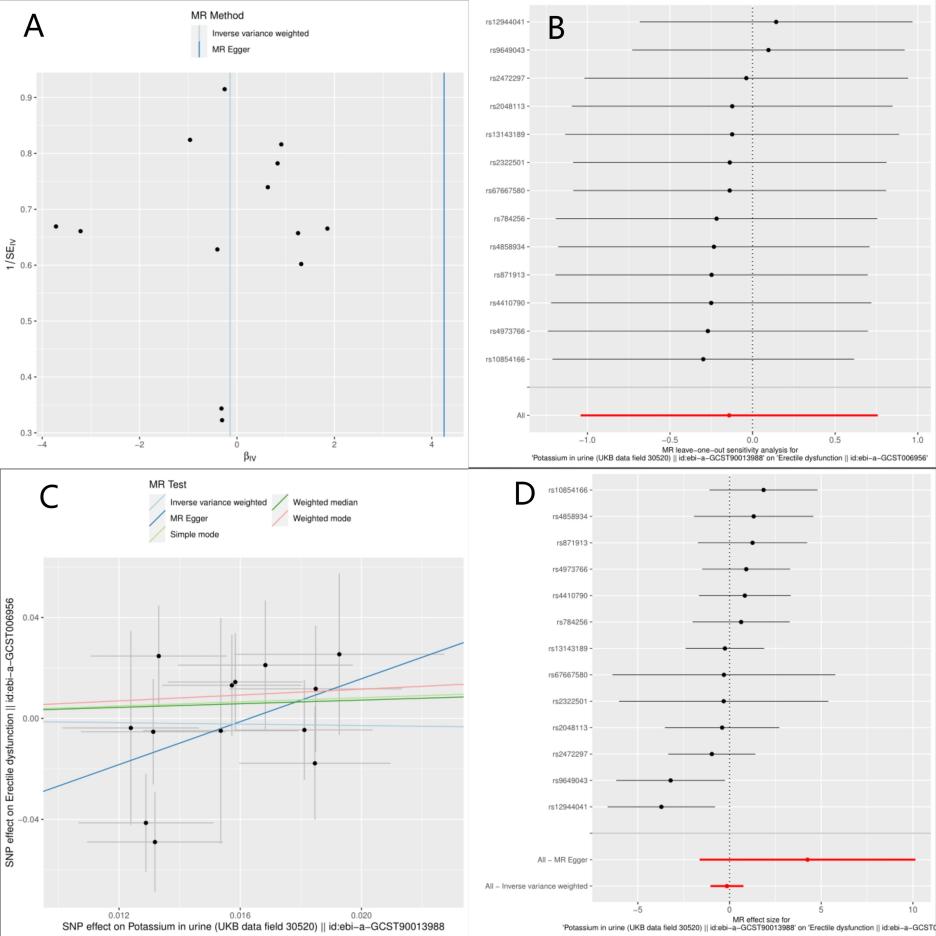


**Figure. 10 (A)Funnel plot; (B)Sensitivity analysis; (C)Scatter plot; (D)Forest plot of the effect of Potassium in urine on erectile dysfunction.**


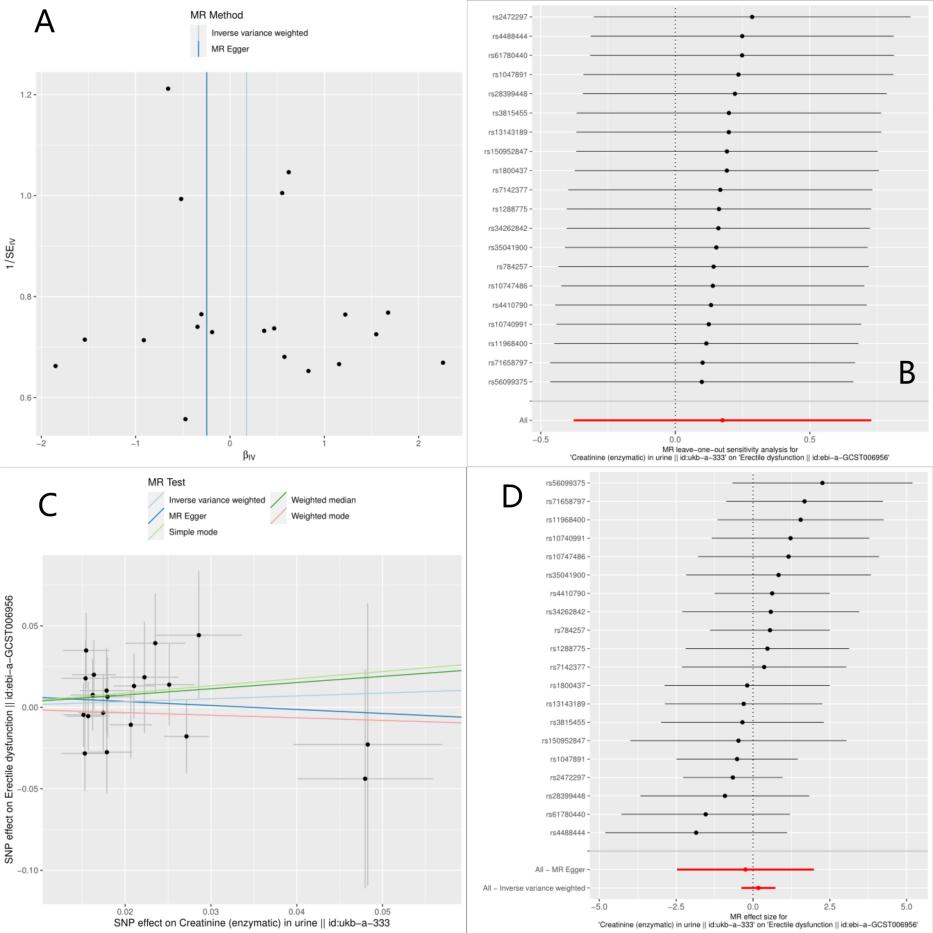


**Figure. 11 (A)Funnel plot; (B)Sensitivity analysis; (C)Scatter plot; (D)Forest plot of the effect of Creatinine (enzymatic) in urine on erectile dysfunction.**


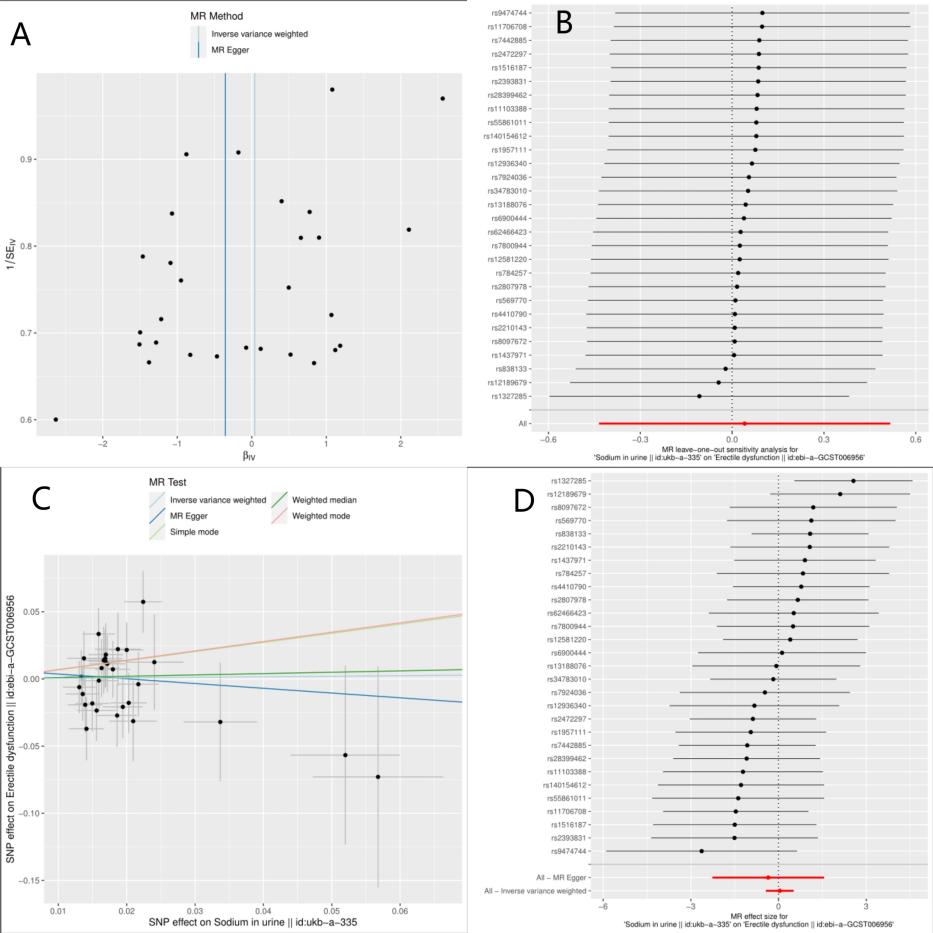


**Figure. 12 (A)Funnel plot; (B)Sensitivity analysis; (C)Scatter plot; (D)Forest plot of the effect of Sodium in urine on erectile dysfunction.**


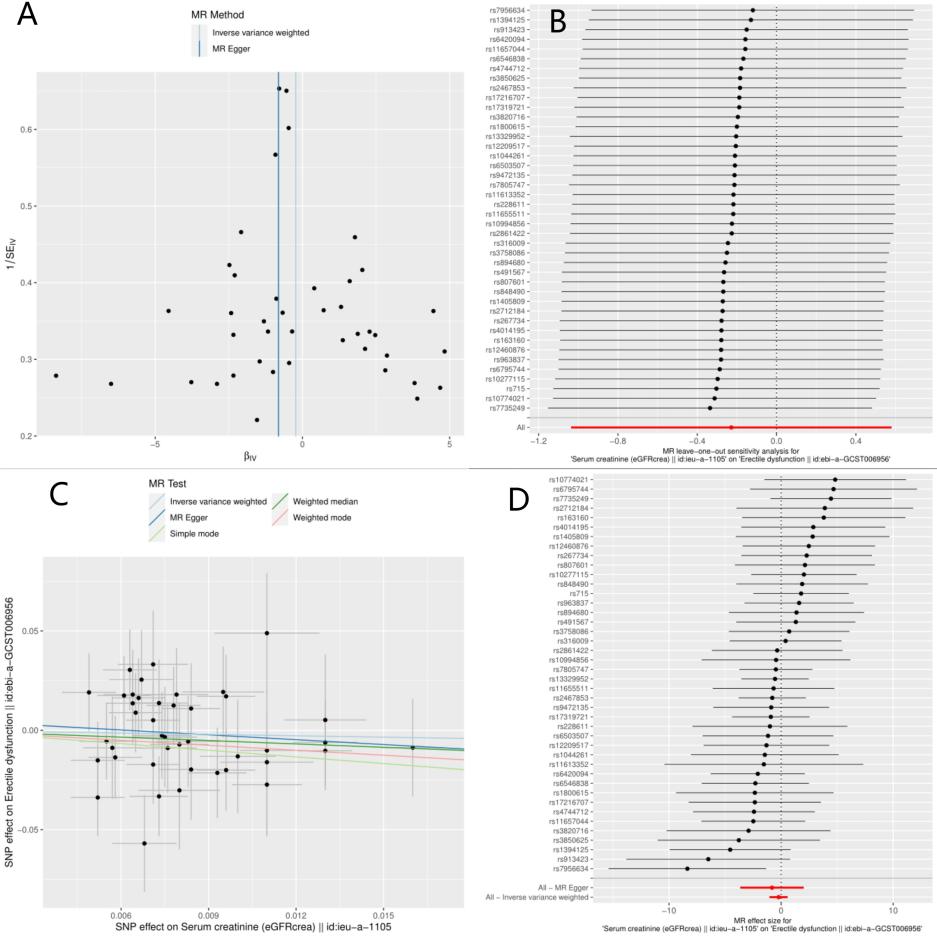


**Figure. 13 (A)Funnel plot; (B)Sensitivity analysis; (C)Scatter plot; (D)Forest plot of the effect of Serum creatinine (eGFRcrea) on erectile dysfunction.**


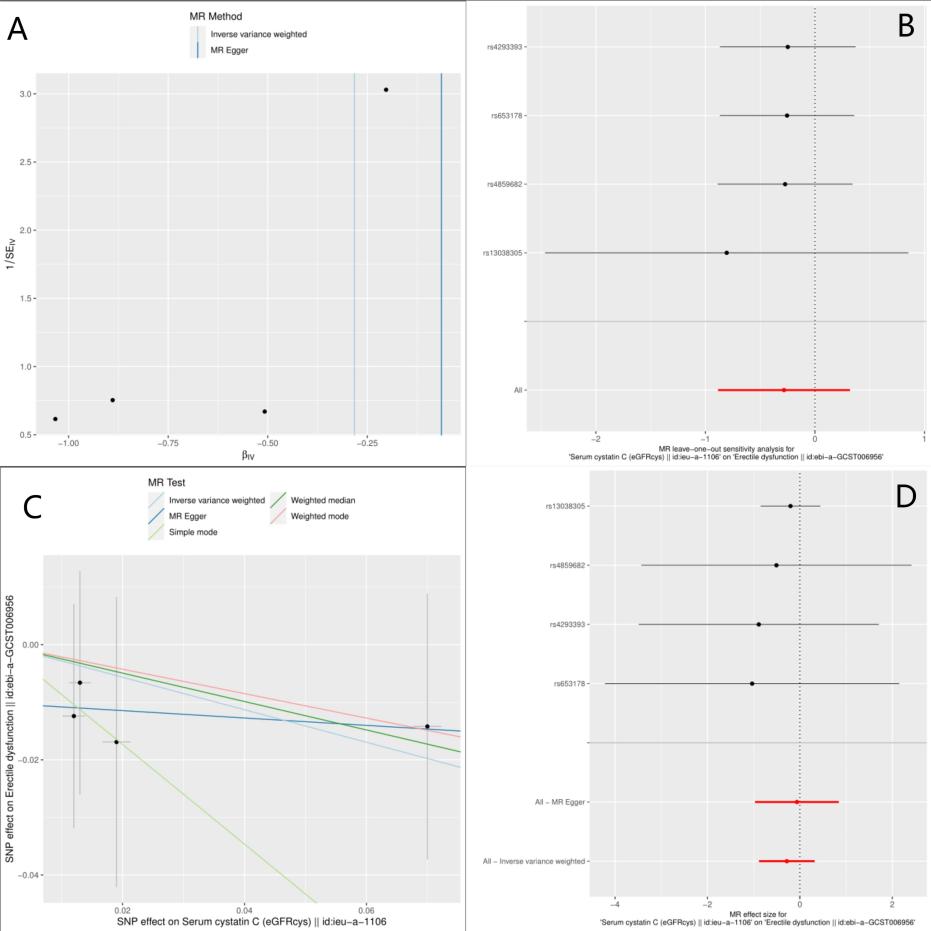


**Figure. 14 (A)Funnel plot; (B)Sensitivity analysis; (C)Scatter plot; (D)Forest plot of the effect of Serum cystatin C (eGFRcys) on erectile dysfunction.**


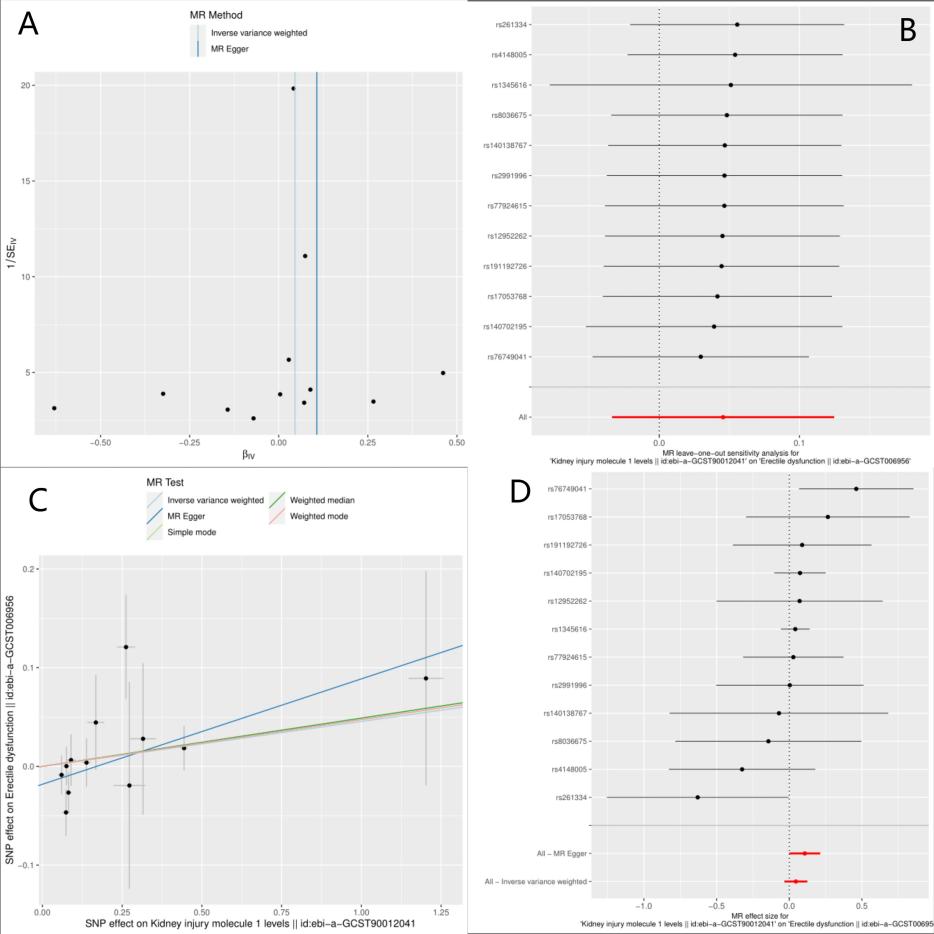


**Figure. 15 (A)Funnel plot; (B)Sensitivity analysis; (C)Scatter plot; (D)Forest plot of the effect of Kidney injury molecule 1 levels on erectile dysfunction.**

**TABLE 3 SNPs excluded due to the presence of confounding factors.**

**Prostate cancer**

rs11263763 ; rs11666569 ; rs12570611 ; rs1634741 ; rs2242652

**IgA nephropathy**

rs2524075

**Urinary albumin excretion**

rs10207567;rs11264327; rs4665972; rs702634

**Potassium in urine**

rs7766356

**Sodium in urine**

rs11642015; rs1260326; rs13383034

**Serum creatinine (eGFRcrea)**

rs1260326;rs1260326

**Serum cystatin C (eGFRcys)**

rs4714704
